# Instrumental Validity of the Motion Detection Accuracy of a Smartphone-Based Training Game

**DOI:** 10.3390/ijerph18168410

**Published:** 2021-08-09

**Authors:** Bernhard Guggenberger, Andreas J. Jocham, Birgit Jocham, Alexander Nischelwitzer, Helmut Ritschl

**Affiliations:** 1 Department of Health Studies, University of Applied Sciences JOANNEUM, 8020 Graz, Austria; andreas.jocham@fh-joanneum.at (A.J.J.); birgit.jocham@fh-joanneum.at (B.J.); helmut.ritschl@fh-joanneum.at (H.R.); 2 Department of Applied Computer Sciences, University of Applied Sciences JOANNEUM, 8020 Graz, Austria; alexander.nischelwitzer@fh-joanneum.at

**Keywords:** telerehabilitation, physiotherapy, augmented reality, low-cost, training game, accuracy, smartphone

## Abstract

Demographic changes associated with an expanding and aging population will lead to an increasing number of orthopedic surgeries, such as joint replacements. To support patients’ home exercise programs after total hip replacement and completing subsequent inpatient rehabilitation, a low-cost, smartphone-based augmented reality training game (TG) was developed. To evaluate its motion detection accuracy, data from 30 healthy participants were recorded while using the TG. A 3D motion analysis system served as reference. The TG showed differences of 18.03 mm to 24.98 mm along the anatomical axes. Surveying the main movement direction of the implemented exercises (squats, step-ups, side-steps), differences between 10.13 mm to 24.59 mm were measured. In summary, the accuracy of the TG’s motion detection is sufficient for use in exergames and to quantify progress in patients’ performance. Considering the findings of this study, the presented exer-game approach has potential as a low-cost, easily accessible support for patients in their home exercise program.

## 1. Introduction

Demographic changes and the associated increase in the age of the population raise several new challenges for the health care system [1]. A significant increase in the total number of hospitalizations is expected in the coming years [2]. Part of this increasing number of hospitalizations is due to the growing number of patients with osteoarthritic degenerative disorders and the associated medical interventions. In the United States of America, total hip arthroplasties (THA) are predicted to increase by over 280% and knee replacements by over 400% by 2040 [3]. For the countries of the Organisation for Economic Co-operation and Development, it is assumed that THA will increase from 1.8 million per year in 2015 to 2.6–2.9 million per year in 2050 [4]. However, with surgery and subsequent inpatient rehabilitation, patients are not sufficiently rehabilitated. Studies have shown that gait abnormalities can still be detected up to one year after implantation of a hip prosthesis [5,6,7]. Considering the increasing number of THA and the need for more optimal rehabilitation strategies, it is necessary to investigate innovative approaches focused on supporting patients’ training beyond the inpatient rehabilitation period [8]. In addition to the therapeutic and sport science concepts currently used, exer-games have recently emerged as a promising alternative based on their potential for making training more varied, exciting and thus more motivating [9,10]. Exer-games are computer games that rely on control through active movement of the users [11].

In addition to game-based approaches, new technologies such as augmented or virtual reality have already been investigated for their use in therapeutic applications [12,13]. Unlike virtual reality (VR), where a complete virtual environment is created, in augmented reality (AR) the real environment is augmented with virtual objects. Special headsets, so called head-mounted displays (HMDs), are commonly used to project virtual objects into the user’s field of view [14].

To address the challenges which occur due to this demographic change, an AR training game (TG) was developed in the project TRIMOTEP [15,16]. The TG was intended for patients in their home training program after THA and completing subsequent inpatient rehabilitation (this corresponds to rehabilitation phase III and IV [17]). The aim of the project TRIMOTEP was to develop an application with low acquisition costs, adaptive training scenarios and easy handling. For this reason, an HMD was used, which uses a smartphone as the central computing unit. In these, the smartphone’s display, sensor technology and computing power are used in combination with a headset to implement AR [18]. To ensure the acceptance of the TG by the end users, patients and physiotherapists were included in the development process via focus groups and expert interviews. The intention of the project was to develop a TG to train coordination, mobility and lower limb strength through body weight exercises in an AR environment. The smartphone’s sensors are used to detect the patients’ movements and recognize predefined exercises. The game content is projected from the smartphone display via Headset into the patient’s field of vision. In the scope of AR applications, no comparable cost-effective system exists at the time of the development of this application. Other AR technologies have been investigated in different medical contexts [19,20]. Currently, the research on smartphone-based AR therapy applications is scarce, and it does not refer particularly to HMD use [21].

During the project TRIMOTEP, the motion detection of the TG had already been tested in a previous usability study and is sufficient to determine which of the given exercises is performed [18]. However, although the TG can detect the given exercises, it is unclear how accurately the overall movements (the motions while using the TG) can be detected. This would be important, for example, to identify incorrect exercise execution or progression in performance (e.g., larger amplitude during squat). Therefore, in this study, the accuracy of the overall motion detection (position and rotational trajectory detection) is investigated. To examine how accurately the motion detection works at specific events, the maximum amplitudes for the implemented exercises of the TG are evaluated. The measurements were carried out in a motion analysis laboratory, with a group of healthy subjects.

## 2. Materials and Methods

To assess the motion detection accuracy of the smartphone-based TG, comparative measurements were performed with healthy individuals. The recorded motions of the TG were compared with those of a high-resolution optoelectronic 3D motion analysis system (3DMAS). For this purpose, 30 participants performed two series of the exercises included in the TG (squat, side-step and step-up). The motion data was recorded synchronously by the TG and the 3DMAS.

### 2.1. Subjects

The study was approved by the Ethics Committee of the Medical University of Graz (IRB00002556) and the volunteers were recruited via email from employees and students of FH JOANNEUM University of Applied Sciences in Graz. Inclusion criteria were legal capacity according to the Adult Protection Act and signing an informed consent. Exclusion criteria for participation were acute orthopedic conditions, acute pain and the presence of medical conditions that could affect the use of the TG. In addition, previously known problems with cyber sickness, such as nausea and vertigo, while using AR or VR applications [22], were also considered to be part of the exclusion criteria. The sample size for accuracy assessment of other motion detection approaches ranged from 4 to 26 participants and showed a strong correlation between the measurement systems [23,24,25,26]. Assuming a strong correlation and with an alpha error of 0.05 and a beta error of 0.2, a sample size of 29 subjects was calculated [27]. Since we wanted to collect at least one exercise amplitude from each subject, we aimed to include 30 subjects.

A total of 30 subjects fulfilled the inclusion criteria and were enrolled in the study. Table 1 shows detailed information of the study population.

### 2.2. Exer-Game

**Figure 1:**
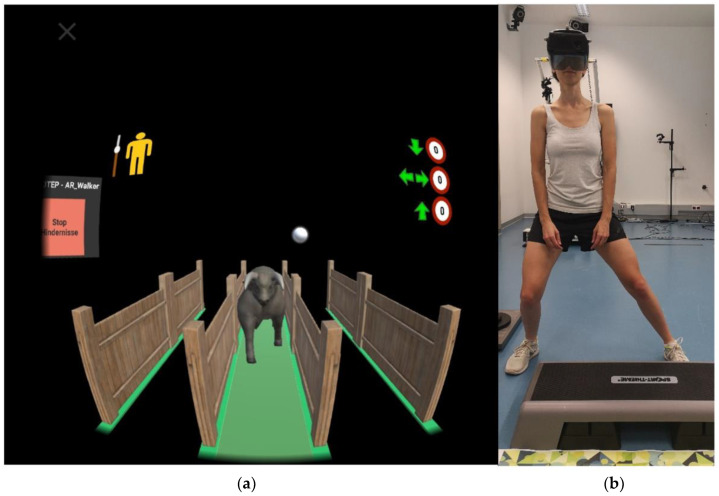
Performing the training game. (**a**) View of the user. The image shows the virtual objects which are placed in the user’s field of view. When using the training game, users see their surroundings instead of the black background. (**b**) The user, wearing the head mounted display, reacts to the obstacle (a bull) and performs a side-step.

The TG (AR Walker application version 1.5 (University of Applied Sciences JOANNEUM, Graz, Austria)) was developed as part of the project TRIMOTEP, to support patients’ training at home after THA. The aim of the game is to avoid contact with obstacles by performing predefined exercises. For each obstacle, there is a defined exercise that has to be learned before using the game in home training. This instruction should take place in a physiotherapy session. The exercises implemented in the software are widely used in rehabilitation for patients after THA and have been selected through expert interviews and focus groups with physiotherapists. The exercises include squats (normal squatting movement), side-steps (clear step sideways in the frontal plane) and step-ups (step-up on a step board) [28]. The TG is based on an AR environment, which consists of three tracks separated by fences, on which randomly generated farm animals (obstacles) approach the users (Figure 1). Therapists have the possibility to obtain information about the extent and the success of the training sessions via reporting in the back end of the software. A current version of the TG application can be downloaded at https://trimotep.fh-joanneum.at/exer-game-ar_walker/ (accessed on 23 July 2021).

The AR HMD consists of a Haori Mirror-headset (Shenzhen Haori Technology Co., Ltd., Shenzhen, China) and an Android smartphone, that fulfils the requirements of ARCore technology. In the present study, a Samsung Galaxy S9 (Samsung Electronics Co., Ltd., Suwon, Korea) and a Huawei P20 smartphone (Huawei Technologies Co., Ltd., Shenzhen, China) were used. No further sensor technology was used for implementation of AR environment and for motion detection.

To track the motions of these exercises and to generate the corresponding AR images, the Google ARCore API (application programming interface (Google LLC, Mountain View, CA, USA)) was used. For this purpose, ARCore implements the smartphone’s built-in inertial measurement unit (IMU) and the integrated front camera. The IMU provides translational and rotational acceleration data. By calibrating the system, using camera images of the room, ARCore detects feature points, which are used as reference for positioning virtual objects and in combination with the IMU data for motion tracking [29]. To improve the detection of the feature points in the room, a sheet with a high-contrast pattern was positioned on the floor, in front of the subjects. During the calibration process of the TG, the users were asked to look at the ground. The feature points detected on the ground and the data from the IMU were used to calculate the distance between the HMD and the ground (approximately the height of the user). The exercises implemented in the training game were defined by threshold values, adapted to the individual users by the determined height. When users reached the thresholds of an exercise, they receive visual and auditory feedback [18]. The exercises were selected taking into account that they differ in their primary direction of movement.

### 2.3. Reference System

**Figure 2:**
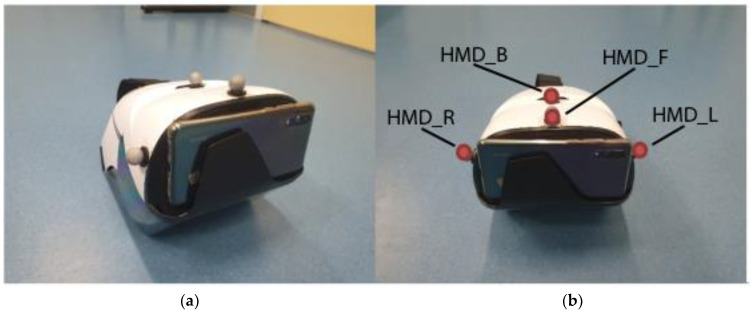
Marker Placement on the Head Mounted Display consisting of a headset and a smartphone. (**a**) Shows marker placed on the head mounted display. (**b**) The four markers are highlighted red and labelled as in the used marker model.

An optoelectronic 3DMAS (Vicon Motion Systems), that collects motion data simultaneously to the ARCore TG, was used as reference. The system consisted of 10 MX3+ cameras and was operated with the Nexus 2.10.1 software (Vicon Motion Systems, Yarnton, UK). The recordings were taken with a recording frequency of 120 Hz. To capture the executed motions, while using the TG, four reflective markers were attached to the HMD. Figure 2 shows the marker placement on the HMD and the assigned names as defined in the marker model.

Currently, optoelectronic 3DMAS are commonly used in laboratory-based motion analysis [30]. They are characterized by an accuracy of less than one millimeter error in the detection of markers [31,32]. In addition to their use in motion analysis, 3DMAS are also used as a gold standard in verifying the accuracy of new methods of motion detection [23,24,31,33,34,35].

### 2.4. Measurement Protocol

Before the measurements, the structure of the TG was explained to the participants and they were shown how to start and stop the individual training sessions. Furthermore, the three exercises included in the TG were explained and demonstrated. In addition, the participants were asked whether they would like to practice the exercises. The measurement was taken as soon as the subjects felt sufficiently prepared to perform the exercises.

The measurement consisted of two parts (further called trial 1 and trial 2), which are shown in Table 2. In trial 1, the exercises were performed by all subjects according to a standardized protocol. In trial 2, the subjects followed the instructions of the TG with exercises in a random order for about one minute.

For both trials, the TG was restarted and the calibration of the TG was carried out again. After the start of the measurement, a defined synchronization movement (nodding of the head five times) was performed before the respective task. This was required in order to synchronize the TG and the 3DMAS using a cross-correlation [36].

**Figure 3:**
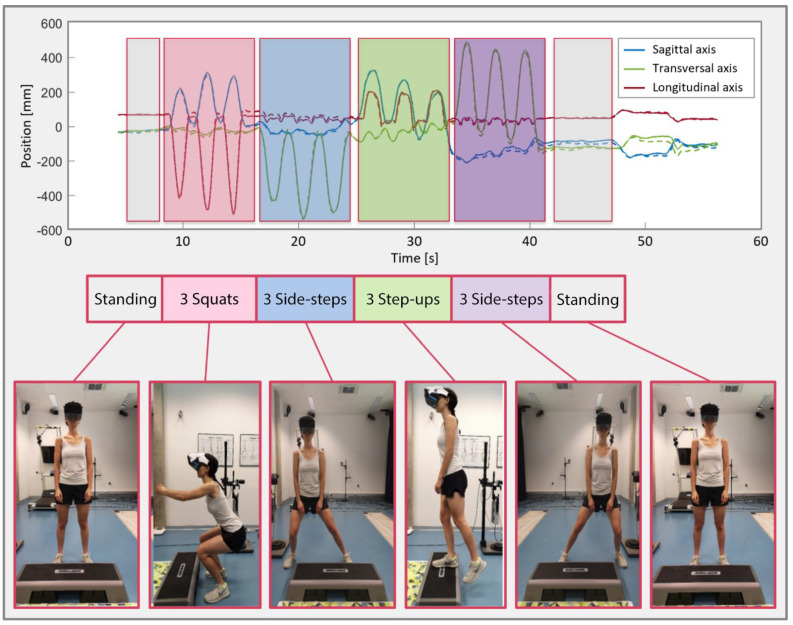
Example of the position trajectories of all spatial planes of one subject recorded with the TG (dashed line) and Table 3. DMAS (solid line) during Trial 1 with an illustration of the procedure. The dashed line is only visible in the case of larger differences between the two measurement systems.

Figure 3 shows the position trajectories of one subject recorded with the TG and the 3DMAS, as well as an illustration of the procedure of Trial 1.

### 2.5. Analysis and Statistics

The initial processing of the 3DMAS data was performed by using Vicon Nexus 2.10.1. Further data analysis was carried out using Matlab (v2019a) (The MathWorks, Natick, MA, USA) and SPSS (v26) (IBM, Armonk, New York, NY, USA). To examine the trajectories and angular curves, respectively, the mean absolute difference (MAD) and the root mean square error (RMSE) were calculated [31,37].

To evaluate the accuracy of the exercises, the axis with the largest amplitude was defined for each exercise. The transversal axis was defined for the side-step, whereas the longitudinal axis was defined for the squat and the step-up. The maximum amplitude of the two systems was calculated independently of time along this axis for the related exercise. The analysis of these maxima was performed by means of bivariate correlation analysis, corresponding Bland-Altman plots, the calculation of the intra-class correlation coefficient (ICC), the mean absolute relative difference (MARD), the MAD and the RMSE [25,31,37,38]. A Kolmogorov-Smirnov test was performed to check normal distribution of the exercise amplitudes [39]. The alpha error was assumed to be 0.05.

From the 60 acquired trials, 59 were used in the data analysis. Due to data corruption of unknown origin, the data sample of one measurement from the TG was not used.

## 3. Results

### 3.1. Position Trajectories

**Figure 4:**
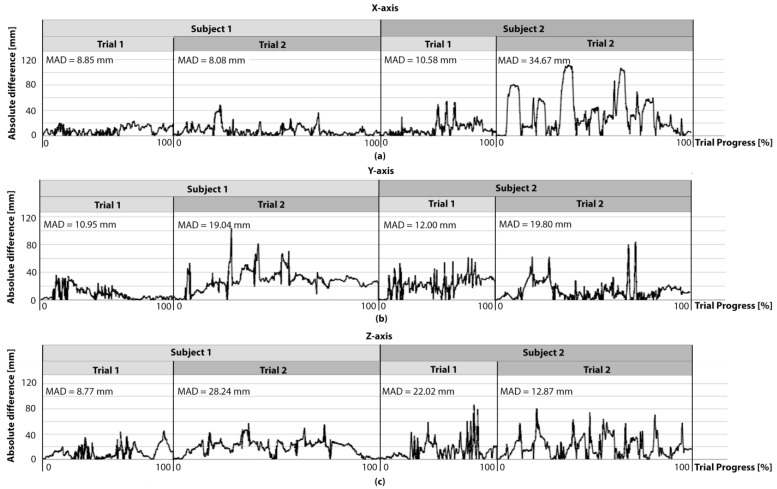
Plots of the absolute difference over time for the two trials of two representative subjects. The progress in one trial is shown as percentage of the duration. Therefore, 0% marks the start and 100% the end of the trial. For each trial the corresponding mean absolute difference (MAD) is shown. (**a**) Shows the absolute difference over time for the trials of two representative subjects along the sagittal axis. (**b**) Shows the absolute difference over time for the trials of two representative subjects along the transversal axis. (**c**) Shows the absolute difference over time for the trials of two representative subjects along the longitudinal axis.

In summary, position and rotational trajectories in 59 trials overall (trial 1 and trial 2 of the subjects combined) with a total duration of 68 min and 52 s were analyzed. Figure 4 shows the absolute difference along the three axes over time for the trials of two representative subjects.

Table 3 shows the results for the MAD and the RMSE for the corresponding axis trajectory.

### 3.2. Rotational Trajectories

For the rotational trajectories the same 59 trials (trial 1 and trial 2 of the subjects combined) as for the position trajectories were analyzed. Table 4 shows the results for the MAD and the RMSE for the corresponding rotational trajectory.

### 3.3. Exercise Amplitudes

From trial 1, 174 side-steps, 90 squats and 87 step-ups could be identified and examined. The Kolmogorov-Smirnov test did not show normal distribution for the data of the side-steps and step-ups (*p* < 0.05). Therefore, for bivariate correlation analysis Spearman’s Rho was calculated for all exercises. It showed a strong correlation between the measurements of the two systems for the amplitude of 0.938 (*p* < 0.001) for the step-up, 0.994 (*p* < 0.001) for the squat and 0.952 (*p* < 0.05) for the side-step. The ICC (95% confidence interval; *p*-value) showed a very good internal correlation with a value of 0.964 (0.899–0.983; *p* < 0.001) for the step-up, 0.998 (0.997–0.999; *p* < 0.001) for the squat and 0.982 (0.976–0.987; *p* < 0.001) for the side-step.

**Figure 5:**
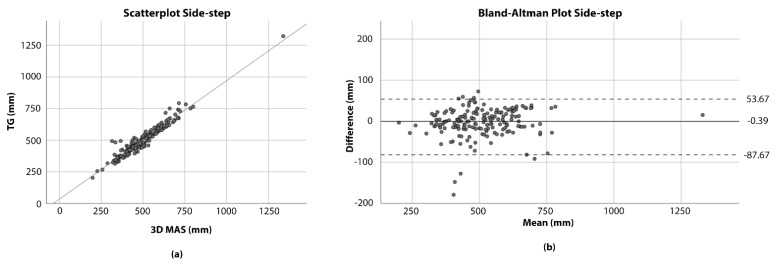

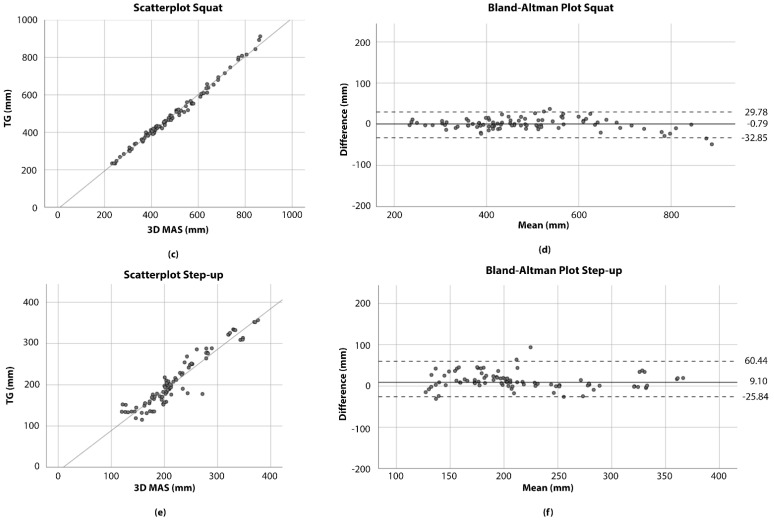
Scatterplots and Bland-Altman plots for the examined exercises. In the Bland-Altman plots the dotted lines represent the limits of agreement and the bold line the median. (**a**) Shows the scatter plot for the side-step. (**b**) Shows the Bland-Altman plots for the side-step. (**c**) Shows the scatter plot for the squat. (**d**) Shows the Bland-Altman plots for the squat. (**e**) Shows the scatter plot for the step-up. (**f**) Shows the Bland-Altman plots for the step-up.

As the Kolmogorov-Smirnov test did not show normal distribution for the measurement differences between the systems of the step-ups (*p* < 0.05), a variant of the Bland-Altman plot for non-parametric data was used for all exercises. For this, the median was calculated and the limits of agreement were set at the 2.5th and 97.5th percentiles. Figure 5 shows the scatterplots and the corresponding Bland-Altman plots for the three exercises squat, side-step and step-up.

Table 5 shows the MAD, the RMSE and the MARD for the squat, side-step and step-up along the main movement axis as described in the methods.

## 4. Discussion

A mean difference of 0.47° to 0.73° could be identified for the rotational trajectories. In comparison, investigations of the performance of an Oculus Rift-based application for measuring the range of motion of the cervical spine revealed larger differences of 2.3° for the flexion angle, 5.4° for the lateral flexion angle and 3.7° for the rotation angle [40]. When comparing these measurement differences, it should be considered that, while executing the exercises correctly during the TG, the head is held relatively still in a constant line of vision. Consequently, only minor absolute changes (mostly below 10 degrees) in the rotational trajectories occurred during the measurements in this study. In contrast, distances of 100 cm or more were usually covered during the linear movements. It is therefore conceivable that the deviation in measurement accuracy is related to the extent of the respective movement component. In future studies, movement sequences with larger rotational movement could provide more information about the accuracy of the rotational trajectories.

For the translational movements, averaged differences of 18.03 mm to 24.98 mm and a maximum RMSE of 35 mm were measured along the different axes. Comparable studies using the Microsoft Kinect for motion capturing, which was used in several studies for exer-game applications, showed a difference of 76 mm to 100 mm for the key points [41,42].

The results show that the presented approach provides comparable accuracy in motion detection to other exer-game systems investigated and thus seems suitable for this domain, with regard to the translational trajectories. However, the accuracy of the measurement may not be sufficient to detect subtle movement differences in the same movement sequences, which could be caused by small changes in patients’ performance, but also by incorrect execution of the exercises (e.g., quality of squat movement). Subsequently, it should be investigated whether this approach can discriminate between predefined exercises whose movement sequences are more similar in their main direction of motion, such as squats and lunges.

For the amplitudes of the exercises, the MARD lies between 2% and 8%, regardless of the size of the measured values. Comparing these results with those of other studies, which investigated IMU-based systems, slightly lower measurement differences in the range of 2° are found [43]. Accuracies of just 1% to 5% have been demonstrated for motion capturing with smartphones [44]. Therefore, it seems that the featured hardware has potential for further improvement of the measurement accuracy beyond the current level. Looking at the accuracy achieved with the current state of the TG, the maximum deflections of exercise amplitudes can be measured with mean differences ranging from 10.13 mm to 24.59 mm. Furthermore, the amplitudes show a strong correlation and a very good internal correlation between the TG and the reference system. The measured accuracy should therefore allow the detection of larger changes in the range of motion of an exercise (e.g., larger amplitude during squat) and thus to determine progression of the patients’ performance. This offers possibilities of providing visual feedback to the patients and thus to integrate additional motivational aspects into the TG.

When evaluating changes in the execution of movements, it should be considered that the TG scans its position with a frequency of 30 Hz [29]. This presents a certain challenge for the recording of fast movement sequences, such as jumps. Nevertheless, the measured accuracies of the exercise amplitudes show that positions at specific events (e.g., lowest position during a squat) can be recorded with comparable accuracy to the overall position trajectories.

The comparison of the trials represented in Figure 4 shows a varying level of inter- and intraindividual (between two trials) accuracy. This is also reflected in the standard deviation of the average measurement difference. These deviations cannot be explained by the different prior knowledge regarding AR of the participants. One reason for the fluctuating quality of detection accuracy may be that only few feature points for orientation were recognized by the TG, since they are important for the motion detection [29]. An empty room with uniform floor and wall color turned out to be disadvantageous for precise movement detection. Rooms with many structures (for example shelves or cupboards), which would be expected in patients’ home environment, seem to be more suitable for this process. To provide additional landmarks for the TG, a high-contrast surface was placed in the subjects’ field of view on the floor, in front of the step board. Since ARCore uses the IMU of the smartphone for motion detection, its characteristics can also influence the measurement accuracy. Problems with IMU-based measurement systems can arise due to sensor-specific drift behavior [45]. This involves a drift of measurement values due to an accumulated difference that occurs in the rotational and position trajectories [46]. The individual trials evaluated for this study have a maximum duration of two and a half minutes. As the drift has a larger effect with an increasing period of time, longer measurement durations can lead to higher inaccuracies. Since Google ARCore includes feature points in addition to the IMU data in the determination of position and orientation, there are reference points in space to which movements can be referred, in contrast to the single use of IMU systems. This might reduce the errors caused by sensor drift and minimize the impact of this issue. No relevant drift behavior could be detected during the time period of two and a half minutes for the measurements.

Since the reference system used has a high accuracy and is accepted as the gold standard in motion analysis, it is assumed that the differences recorded in this study originate primarily from the TG itself and not from the 3DMAS.

### 4.1. Limitations

A limitation of this motion analysis approach is that the movement detection is only performed by the sensors of a smartphone attached to the headset. It also must be questioned whether the movements of this HMD represent the actual head movements of the users. Subsequently, it should be investigated whether relative movements between the HMD and the patient’s head may occur. Moreover, the smartphone is positioned at a certain distance in front of the head, and it is unclear whether this has a negative influence on the representation of the head movements. It is also unclear whether and to what extent the measured head movements allow statements about the movements of the remaining body segments and especially the lower extremities. This raises the question as to which movement deviations of the body can be detected even with higher measurement accuracy for the head movement. Hence, how far complex motion sequences of the entire body can be assessed on the basis of the head movements should be investigated.

Another limitation is that only healthy subjects were included in the present study. Therefore, transferability of the reported measurement accuracy to patients with THA or other patient groups is possible only to a limited extent. Furthermore, the experiences with the implemented exercises of the participants were homogeneous. In addition, it is unclear whether movement sequences that are influenced by illness or due to previous medical interventions can be recorded with the same accuracy as measured in the present study. Thus, further accuracy studies should be conducted with patients.

### 4.2. Perspectives

Comparative measurements with the presented approach showed comparable motion detection accuracy to other low-cost systems. It therefore has potential (A) for further exer-game applications, (B) to document exercise and therapy progress and (C) to support tele-rehabilitative home exercise programs. However, it must be noted that, with the approach presented, precise analysis of movements or identification of incorrectly performed exercises, simply by recording head movements, may not be possible. In any case, the TG has the potential to offer a cost-efficient, accessible training support, which is characterized by the widespread use of the hardware (smartphones) and thus the low acquisition costs. Exer-games can motivate patients in their home exercise program, for example, through gamification [9,10]. If exercises can be counted, exercise duration recorded, or exercise completion linked to virtual goals, this already has potential to create motivational factors and support home exercise programs.

Since the software runs on commercially available ARCore-supporting Android smartphones, devices owned by patients could be used for the execution of the TG. The users would only have to be equipped with a compatible headset and a step board. It must be noted that the approach described in the present study only operates with Android smartphones. For the implementation of comparable motion detection approaches on Apple devices, the ARKit may be used [29,47].

Therapeutic exercises are commonly implemented in physiotherapy sessions and as home-based programs for rehabilitation of various diseases [48]. Therefore, the TG may be used as home exercise program in THA patients and in other groups of patients whose treatment involves home exercise programs. Importantly, new movement sequences could be defined in the TG which could expand the current pool of exercises. To increase the segments tracked by the motion detection, the TG could also be supplemented with further low-cost sensor technology, such as single IMUs on certain body segments. Further research could focus on this.

## 5. Conclusions

In summary, the accuracy of the TG’s movement detection is comparable to other low-cost exer-game systems. It seems to be sufficient to differentiate among the implemented exercises and accurate enough to detect progress in patients’ performance (amplitude of movement). One limitation of this system is that the TG records only the motions of the head. The extent to which conclusions can be drawn from this about the motion sequences of the whole body must be investigated in further detail. It is unclear yet how incorrect exercise execution affects the movements the head. Therefore, it is not clear whether it is possible to assess the quality of exercises (correct execution of the exercise) by the motion detection of the TG. Moreover, examinations with larger rotational motions should be carried out. The presented AR exercise game approach is based on smartphones, which are commonly used in the general population. Combining the results of the present study with this fact, the investigated TG seems to have potential for a low-cost and easily accessible approach to support patients in their home exercise program after THA and completed subsequent inpatient rehabilitation.

## Figures and Tables

**Table 1:** Demographics of the study population. Annotation: *N* = sample size, SD = standard deviation.

Demographics	*N* = 30
Age in Years, *N* (SD)	32.0 (±10.1)
Gender, *N* (%)	
Female, absolute (%)	15 (50)
Male, absolute (%)	15 (50)
Height in cm, mean (SD)	175 (±10)
Previous experience with AR/VR applications, *N* (%)	16 (53.3)
Previous experience with squat exercise, *N* (%)	30 (100)
Squat exercise already performed once, *N* (%)	30 (100)
Previous experience with the exercise side-step, *N* (%)	29 (96.7)
Side-step exercise already performed once, *N* (%)	30 (100)
Previous experience with the exercise step-up, *N* (%)	30 (100)
Step-up exercise already performed once, *N* (%)	30 (100)

**Table 2:** Sequence of the measuring parts.

Trial 1	Trial 2
Starting the training game	Starting the training game
Performing synchronization movement	Performing synchronization movement
3 squats	Playing the training game for approximately one minute
3 Side-steps to the right side and back	
3 Step-ups	
3 Side-steps to the left side and back	
Closing the training game	Closing the training game

**Table 3:** Results for the position trajectories along the anatomical axis. Annotation: MAD = mean absolute difference, RMSE = root mean square error.

Axis	MAD	RMSE
Sagittal, mm	18.68 ± 29.7	35.08
Transversal, mm	24.98 ± 26.73	36.59
Longitudinal, mm	18.03 ± 17.66	25.24

**Table 4:** Results for the rotational trajectories.

Rotational Trajectory	MAD	RMSE
Flexion, °	0.48 ± 0.46	0.66
Lateral flexion, °	0.47 ± 0.88	1.00
Rotation, °	0.73 ± 0.96	1.20

**Table 5:** Results of the examined exercises. Annotation: MARD = mean absolute relative difference.

Parameter	Squat	Side-Step	Step-Up
MAD ± SD, mm	10.13 ± 9.4	24.59 ± 24.33	17.31 ± 16.65
RMSE, mm	13.79	34.54	23.96
MARD	0.02	0.05	0.08

## Data Availability

The data presented in this study are openly available in Zenodo at doi:10.5281/zenodo.5024992.
